# Retraction Note: Modelling the interaction between tourism, energy consumption, pollutant emissions and urbanization: renewed evidence from panel VAR

**DOI:** 10.1007/s11356-024-34254-1

**Published:** 2024-07-09

**Authors:** Festus Fatai Adedoyin, Festus Victor Bekun

**Affiliations:** 1 Department of Accounting, Finance and Economics, Bournemouth University, Poole, UK; 2 Faculty of Economics and Administrative Sciences, Istanbul Gelisim University, Istanbul, Turkey; 3 Department of Accounting, Analysis and Audit, School of Economics and Management, South Ural State University, 76, Lenin Aven, Chelyabinsk, Russia 454080

**Retraction Note: Environmental Science and Pollution Research (2020) 27:38881-38900**

10.1007/s11356-020-09869-9

The Publisher has retracted this article in agreement with the Editor-in-Chief. An investigation by the publisher found a number of articles, including this one, with a number of concerns, including but not limited to compromised peer review process, inappropriate or irrelevant references, containing nonstandard phrases or not being in scope of the journal. Based on the investigation's findings the publisher, in consultation with the Editor-in-Chief therefore no longer has confidence in the results and conclusions of this article.

Author Festus Fatai Adedoyin has stated that the authors disagree with this retraction.

